# Nutrition in the Actual COVID-19 Pandemic. A Narrative Review

**DOI:** 10.3390/nu13061924

**Published:** 2021-06-03

**Authors:** Vicente Javier Clemente-Suárez, Domingo Jesús Ramos-Campo, Juan Mielgo-Ayuso, Athanasios A. Dalamitros, Pantelis A. Nikolaidis, Alberto Hormeño-Holgado, Jose Francisco Tornero-Aguilera

**Affiliations:** 1 Faculty of Sports Sciences, Universidad Europea de Madrid, Tajo Street, s/n, 28670 Madrid, Spain; Josefranciso.tornero@universidadeuropea.es; 2 Grupo de Investigación en Cultura, Educación y Sociedad, Universidad de la Costa, 080002 Barranquilla, Colombia; 3 Studies Centre in Applied Combat (CESCA), 45007 Toledo, Spain; ajhh1983@gmail.com; 4 Departamento de Educación, Universidad de Alcalá, 28801 Alcalá de Henares, Spain; domingojesus.ramos@uah.es; 5 Department of Health Sciences, Faculty of Health Sciences, University of Burgos, 09001 Burgos, Spain; jfmielgo@ubu.es; 6 Laboratory of Evaluation of Human Biological Performance, School of Physical Education and Sport Sciences, Aristotle University of Thessaloniki, 54124 Thessaloniki, Greece; dalammi@phed.auth.gr; 7 Exercise Physiology Laboratory, 18450 Nikaia, Greece; pademil@hotmail.com

**Keywords:** COVID-19, nutrition, lockdown, body composition, vitamin, dietary pattern, immunology, physical activity, gut

## Abstract

The pandemic of Coronavirus Disease 2019 (COVID-19) has shocked world health authorities generating a global health crisis. The present study discusses the main finding in nutrition sciences associated with COVID-19 in the literature. We conducted a consensus critical review using primary sources, scientific articles, and secondary bibliographic indexes, databases, and web pages. The method was a narrative literature review of the available literature regarding nutrition interventions and nutrition-related factors during the COVID-19 pandemic. The main search engines used in the present research were PubMed, SciELO, and Google Scholar. We found how the COVID-19 lockdown promoted unhealthy dietary changes and increases in body weight of the population, showing obesity and low physical activity levels as increased risk factors of COVID-19 affection and physiopathology. In addition, hospitalized COVID-19 patients presented malnutrition and deficiencies in vitamin C, D, B12 selenium, iron, omega-3, and medium and long-chain fatty acids highlighting the potential health effect of vitamin C and D interventions. Further investigations are needed to show the complete role and implications of nutrition both in the prevention and in the treatment of patients with COVID-19.

## 1. Background

Originated in Wuhan (China) in December 2019 as a group of unexplained cases of pneumonia caused by the SARS-Cov-2 virus, the World Health Organization (WHO) declared as a pandemic in March 2020, affecting multiple countries around the world and with more than 154 million confirmed cases and more than 3.3 million of deaths [1]. A direct impact on the normal life of people around the world was produced by the actual pandemic with restrictive policies like lockdown, the use of protective masks, and limitations of personal movement [2,3]. The clinical symptomatology varies from asymptomatic to acute respiratory distress syndrome and multi-organ dysfunction [4], being common fever, headache, sore throat, dry cough, fatigue, myalgia, breathlessness, and occasional gastrointestinal symptoms [5]. At the end of the first week of infection, the infection can progress to pneumonia, respiratory failure, and death [4]. This progress is associated with an extreme rise in inflammatory cytokines such as GCSF, IP10, MCP1, MIP1A, IL-2, IL-7, IL-10, and TNFα [4]. Also, eosinophilic cell blood count seems to have a major role in COVID-19 diagnosis and prognosis [6]. Different factors associated with socioeconomic status, age, sex, and race have been investigated concerning COVID-19 [7,8,9,10]. One of them, which plays a fundamental role in the subject’s immune status and its response to disease, is nutrition. Nutritional intervention during the COVID-19 pandemic has revealed different ways of acting with different results. To present the actual knowledge about nutrition in the actual COVID-19 pandemic, we conducted the present research. Then, this study is a narrative review with the aim of collecting published literature and articles regarding dietary patterns, body composition, nutritional deficiencies, vitamin interventions, dietary patterns, nutrition, and physical activity in the COVID-19 pandemic.

### Search Methods and Strategies for Research Identification

The protocol used consisted of a literature search, using primary sources, like scientific articles, and secondary like; bibliographic indexes, databases, and web pages. We used PubMed, Embase, SciELO, Science Direct Scopus, and Web of Science employing MeSH-compliant keywords including COVID-19, Coronavirus 2019, SARS-CoV-2, 2019-nCoV, Nutrition, Diet, Dietary Patterns, Body Compositions, Vitamin, Nutritional, Immunology, Physical Condition, and Physical activity. We used articles published from 1 February 2020 till 13 April 2021, although previous studies were included to explain some information in several points of the review. The following exclusion criteria were used: (i). studies with old data (out of the COVID-19), (ii). present inappropriate topics, being not pertinent to the main focus of the review, (iii). Ph.D. dissertations, conference proceedings, abstracts, unpublished studies, and books. We included all the articles that met the scientific methodological standards and had implications with any of the subsections in which this article is distributed, nutrition and COVID-19. The information treatment was performed by the seven authors of the review. Finally, articles were discussed by the authors to write the present review. A total of 4533 papers were retrieved from the above bibliographic searches, and 2904 papers were removed after the publishing period inclusion criteria. 132 papers were removed as duplicates, and 1472 were rejected after reviewing paper titles and abstracts. The final 157 papers were read to be considered relevant to the search criteria and appropriate for assessing our research objective.

## 2. Dietary Patterns in COVID-19 Pandemic Lockdown

The COVID-19 lockdown has affected the dietary habits and nutritional patterns of the different countries affected. In this way, some discrepancies according to the type and duration of the home confinement, the cultural and social tendencies of the countries, the age of the sample analyzed, and the level of previous obesity have promoted different conclusions. Specifically, in Spain, it has been reported that the diet conducted during the lockdown had a larger energy intake and lower nutritional quality than the pre-COVID-19 eating patterns [11]. This study found that people consume 539 kcal more than the recommendation during the COVID-19 lockdown, presenting the food ingested with lower nutritional quality [11]. In addition, in comparison with the same period in 2019, an increase of 6% of daily intake was found with ingestion of 2509 kcal during the COVID-19 home confinement. In this line, a study conducted in Poland’s population showed how people ate and snacked more during the lockdown, being these tendencies more frequent in overweight and obese individuals [12]. One possible reason to justify this increase in the energy intake could be related to the fact that staying at home, indoor, and working remotely had a direct impact on daily food habits, producing an increase in energy ingestion and the craving for comfort food due to boredom and stress [13]. Governments must take into consideration that these lifestyle changes that modify the diet patterns and quality of foods, like the increased ingestion of high caloric foods and the decreased ingestion of healthy foods like vegetables and fruits, could consequently increase the risk for chronic diseases [14].

Regarding food composition, COVID-19 has modified the frequency of intake of some products and the amount of consumption of others [14,15]. These changes supported the hypothesis that, stays at home and social distancing represent a negative impact on the adherence to healthy dietary patterns [14]. Specifically, a previous study showed that during the lockdown, Spanish people ingested lower amounts of beverages, slightly increased eggs, and red meat ingestion and presented a substantial increase of plant-based foods, like nuts, pasta, rice, or processed vegetables, in comparison with the same period in 2019 [15]. Although these changes were found, the consumption of red meat was still higher than the recommended dietary guidelines, whereas the plant-based food products remained below the recommended range [15]. On the other hand, the dietary patterns of Chinese people during the COVID-19 lockdown also changed, showing a decrease in the frequency of intake of fresh vegetables and fruit, rice, poultry, meat, and soybean products [16].

Interestingly, other research which studied the effect of COVID-19 confinement in an adolescent population of Brazil, Chile, Colombia, Spain, and Italy showed a healthy dietary change [17], increasing the ingestion frequency of vegetables, legumes, and fruit during the lockdown. In this way, the number of adolescents who consume the recommended weekly servings of legumes and fruits during confinement increased by 8% and 7.7%, respectively, in comparison to before confinement moment. Some reasons may justify these patterns changes. First, the legume and fruit sale increased since the beginning of confinement, and second, the people have more time to cook in their homes [17]. Hence, controversial findings have been reported in the recent studies published which analyze the effect of lockdown in dietary patterns. In this way, some possible factors could affect the results reported. For instance, studies analyzed some populations with inherent eating patterns. Additionally, the government of each country established different levels of lockdown with divergences in the severity and the restrictions of the population. These factors may underlie the inconsistent patterns detected in the studies reported [17,18].

Remarkably, within all groups affected by the COVID-19 lockdown, overweight and obese people are particularly impairing their dietary patterns and lifestyles. These population groups are known to display more disruptive eating behaviors, including food consumption without hunger feeling and frequent overeating [18]. In this way, overweight and obese individuals reported eating and snacking more during home confinement [19], which could be explained due to the prolonged stay at home with often unlimited food access. Furthermore, individuals with a higher body mass index (BMI) presented lower frequency in consumption of fruit, vegetables, and legumes during the lockdown and higher consumption of dairy, meat, and fast foods [19]. In addition, lockdown produced modifications in diet patterns of obese children and adolescents, significantly increasing their ingestion of potato chips, sugary drinks, and red meat in comparison to the same period in 2019 [20]. And of course, all these lifestyle changes could produce a final increase in fat mass [21].

The consequences of the unhealthy dietary modifications reported during a short period of lockdown may result in developing unhealthy eating habits in the medium to long term, which could negatively affect their long-term health status [22,23]. The negative emotions as boredom and stress promoted during the lockdown tend to develop these unhealthy eating habits to draw attention away from these negative emotions [24,25]. This fact could be very important during the COVID-19 pandemic because a healthy and balanced diet is an integral part of the personal risk management strategy during the COVID-19 pandemics [26] due to the immunomodulatory effects that some macro-, micro-, and phytonutrients have [27,28]. In addition, it has been shown how nutritional deficiencies were linked to higher host susceptibility to viral infection and a more severe clinical course of diseases [27,28]. Therefore, although a healthy diet does not completely prevent the infection, it may play an intense role in the host response to an infectious agent [19]. Curiously, other unhealthy habits showed a similar pattern to dietary habits. In this way, an increase in alcohol consumption (15%), with a higher tendency to drink more in alcohol addicts’ people, and a rise in smoking frequency during the lockdown has been reported [19]. Therefore, future research is needed to analyze how dietary patterns will evolve after the COVID-19 lockdown and the necessity to recommend a short guideline to promote healthy habits during future possible lockdowns.

## 3. Body Composition and COVID-19 Risk

It has been suggested that an excess of fat or decreased lean mass might affect the physiological functioning of the human body [29]. In this section, the relationship of body composition with COVID-19 was discussed. We focused on the following questions: (a) changes in body composition during the lockdown, (b) changes in body composition correlate (e.g., physical activity and nutrition) during the lockdown, and (c) body composition as a risk factor for COVID-19.

Physical activity was characterized by an abrupt decrease at the beginning of lockdown followed by a small gradual increase during this period [30]. Those with more body fat percentage had a smaller increase in physical activity during lockdown than those with lower body fat percentage [30]. It should be highlighted that body composition was evaluated using proxy measures of fatness such as circumferences [31], bioimpedance analysis [30], or body mass index (BMI) to examine differences among underweight, normal weight, overweight and obese adults [32].

There was evidence that obesity increased COVID-19 risk [33]. For instance, obesity class II presented a greater risk of COVID-19 in adults older than 65 years, whereas BMI shows a linear association with testing positive for this virus in younger than 65 years old adults [34]. Moreover, it has been observed that visceral fat was higher in positive COVID-19 patients requiring intensive care [35]. Also, it has been shown that an increase in visceral fat in COVID-19 patients was related to a higher likelihood of intensive care unit treatment [36]. It has been proposed that body composition on low-dose computed tomography of the chest was a predictor of poor clinical outcome in COVID-19, in a study where body composition was monitored using the ratio of waist circumference per paravertebral muscle circumference [37]. In addition, abdominal adiposity, described by the waist-to-hip circumference ratio, in patients with respiratory symptoms was an independent risk factor for respiratory distress in COVID-19 [38]. Obesity might also influence immunological responses to the virus, inflammatory reaction, metabolic and respiratory distress [39]. An explanation of obesity as a risk factor might be its effect on immunity altering the pathogenesis of acute respiratory distress syndrome and pneumonia [40].

Obesity and morbid obesity were identified as significant risk factors for internal care units (ICU) admission and particularly for invasive mechanical ventilation requirements in COVID-19 [41]. It may function as a clinical predictor for risk stratification models, being the measurement of anthropometric and metabolic parameters in COVID-19 would be crucial, especially for the younger group [42]. Specifically, visceral fat deposition is higher in COVID-19 patients accessing ICU, finding how visceral fat is related to the necessity of intensive medical care [43]. Patients with obesity and elevated BMI should be closely monitored and might need escalation of therapy earlier to avoid unfavorable clinical outcomes [44]. Obesity or BMI should be recommended as important parameters for COVID-19 risk assessment, and Obese patients with COVID-19 should be treated as a higher risk population [45,46].

In the context of energy balance, the detrimental changes in body composition during lockdown should be attributed to corresponding changes in physical activity and nutrition, reflecting a decreased energy expenditure and increased caloric ingestion [47]. In addition, it was demonstrated how obesity could negatively influence the efficacy of the vaccine in rabies, tetanus, hepatitis B, and influenza, so in the current vaccination situation, it could also be another limitation for the efficacy of the COVID-19 vaccine [48,49,50,51].

## 4. Nutritional Deficiencies of COVID-19 Patients

The relationship between nutrition and the immune system is well known, so much attention is being paid to its role in COVID-19 [52]. In this sense, although it does not appear to be a cure for COVID-19, healthy eating patterns seem to optimize the immune system function and contribute to a lower probability of COVID-19 contagion and to recover better in those who have suffered it [7]. This fact is especially important considering the healthcare overload due to the pandemic, highlighting the importance of nutrition in the correct general health and immune response of the population. Specifically, the Mediterranean diet and other dietary strategies that reduce inflammation and the risk of chronic disease could reduce the risk of severe disease and mortality from COVID-19 [53]. In addition, certain nutrients such as vitamins A, B related vitamins (folic acid, vitamins B6 and B12), vitamin D, vitamin C, and the minerals Se, Fe, Cu, and Zn, are important for proper immune function [54]. Therefore, it is plausible to believe that deficiencies and a suboptimal nutritional status of these micronutrients could potentially favor the spread of COVID-19 by reducing resistance to infection and reinfection.

Surely the most studied micronutrient in relation to COVID-19 is vitamin D because the vitamin D receptor is expressed in almost all types of cells of the immune system (for example, B and T lymphocytes, dendritic cells, macrophages, and monocytes) [55]. Consequently, the correct immune system function will depend on the correct bioavailability of vitamin D from these cells. Although vitamin D deficiency has not been associated with an increased likelihood of COVID-19 infection, a positive association has been observed between vitamin D deficiency and disease severity [56]. Thus, the most severe cases of COVID-19 have shown 64% more vitamin D deficiency than mild cases. Likewise, insufficient vitamin D levels increase the probability of hospitalization and mortality by COVID-19 [56].

On the other hand, although there is sufficient evidence to indicate the relationship that other micronutrients have on the immune system [57], there is little research that links these with the likelihood and/or severity of COVID-19 [58,59]. However, the data shown indicate that, while the hospitalized patient is not usually deficient in vitamins B1 and B12 or zinc, the vast majority can reveal at least one nutrient deficiency [59]. Specifically, 42% of patients hospitalized for COVID-19 have presented selenium deficiency, 6% of vitamin B6, and 4% of folate [59]. These results suggest that together with vitamin D deficiency, selenium deficiency could decrease immune defenses against COVID-19 and cause a progression to serious disease. In addition, selenium is involved in the differentiation, proliferation, and normal function of many innate immune system cells. Additionally, selenium is also crucial in the adaptive response, aiding in the production and development of antibodies [60]. However, more precise and large-scale studies are needed to confirm these results.

Another micronutrient whose influence on respiratory tract infections, this type of pathology is one of the most serious in COVID-19 patients due to its antioxidant role is vitamin C [61]. In addition, vitamin C presented other pleiotropic and important functions in the immune function, including the regulation of hundreds of genes in immune cells [62]. Serum vitamin C levels have been observed to be low in most critically ill COVID-19 patients. In addition, along with age, vitamin C appears to be a codependent risk factor for mortality from COVID-19 [63].

There is no research that directly relates the levels of other micronutrients in the prevention and treatment of COVID-19, although they play an important role in immunity [64]. However, an ecological study in which the relationship between the nutritional status of the country population and the epidemiological data of COVID-19 in 10 European countries revelated that the suboptimal consumption of iron and vitamin B12 correlated with a higher incidence or mortality of COVID-19 [64]. While iron participates in various immune processes and is an essential component for some enzymes involved in crucial immune cell activities [65], low levels of B12 elevate methylmalonic acid and homocysteine, resulting in increased inflammation, and increased production of reactive oxygen species, and oxidative stress [66].

Another important aspect to consider is the nutritional status of patients with a prolonged stay in hospitals and especially in intensive care (>5 days) [67]. In this sense, despite a personalized diet that includes supplementation with vitamin D and trace elements during the hospital stay, the prevalence of malnutrition among hospitalized patients with COVID-19 is approximately 50.0% with age independence [68]. Malnutrition during hospitalization for COVID-19 is the product of increased energy expenditure associated with ventilatory work during a severe respiratory infection that causes an inflammatory syndrome and hypercatabolism and of a greatly reduced food intake caused by different factors such as respiratory distress, anosmia, ageusia, and digestive symptoms (anorexia, diarrhea, vomiting or abdominal pain) [67]. Thus, approximately 40% of patients suffer a weight loss of ≥5% during their hospitalization that defines cachexia [69]. Furthermore, biologically these patients present, among others, hypoalbuminemia, hypoproteinemia, hypocalcemia, anemia, hypomagnesemia, and vitamin D deficiency [67]. This fact is exacerbated by immobilization and could also contribute significantly to muscle atrophy and sarcopenia in COVID-19 [70]. In this sense, although there are no specific treatments for use on patients who have been hospitalized for COVID-19, treatments should focus on nutritional support and rehabilitation exercise whenever possible to prevent long-term disability due to acute illness due to COVID-19 [69].

The relationship between nutrition and COVID-19 disease is becoming clearer every day. Although they are not decisive in the contagion of COVID-19, deficiency states of some nutrients are a prognostic factor of the disease. Specifically, deficient states of vitamin C, D, and selenium, as well as the suboptimal consumption of iron and vitamin B12, have been shown to raise the probability of hospitalization and mortality from COVID-19. On the other hand, despite receiving individualized nutrition during their stay, most patients who have suffered a hospital stay of more than five days have presented a state of malnutrition/cachexia upon leaving the hospital.

Therefore, public health organizations are encouraged to promote population nutritional strategies that include supplementation or foods rich in nutrients related to the prognosis of COVID-19, especially in vulnerable populations such as the elderly, to maintain optimal immune function, especially in times of infections such as COVID-19 [71]. In addition, it would be advisable to monitor COVID-19 surviving patients for a period of 3 to 6 months in which anthropometric, clinical, and laboratory evaluations are carried out to guarantee adequate recovery [72].

## 5. Vitamin Intervention in COVID-19 Pandemic

Until the widespread vaccination or a specific drug treatment against COVID-19 is approved, a great deal of attention has been focused on nutritional interventions as a prophylaxis and treatment potential [73]. A great list of minerals, natural products (not processed), probiotics and prebiotics, omega-3 fatty acids, and mostly vitamins A to E have gained much attention since the onset of the pandemic, considering their anti-inflammatory, antioxidant and immune-boosting role and evidence from previous clinical trials showing potential benefits against respiratory infections [74].

Vitamin D is an essential nutrient, a fat-soluble prohormone steroid that has endocrine, paracrine, and autocrine functions [75], that has been positively associated with lower severity symptoms in COVID-19 elderly patients [76] through different mechanisms [77]; however, considering the mortality rates, the existing body of knowledge has not reached a consensus [78]. Generally, vitamin D deficiency seems to co-exist in COVID-19 patients [77]. In any case, increasing the levels of vitamin D-deficient patients or high-risk populations at the optimal levels (75–125/mL) is commonly suggested [79], although defining the deficient and sufficient levels is a matter of much debate [80]. As such, the recommended dose, based on the measurement of serum 25-hydroxyvitamin D, shows a great inter-individual variation depending on the chronological age, the incubation period (early or late), and the pre-existing comorbidities [81]. Moreover, physiological (e.g., skin tone, body mass index), environmental (e.g., pollution), and seasonal factors, as well as the consumed form (D2 or D3) [82], have been examined. The co-supplementation with L-cysteine [83] or magnesium and vitamin B [84] has also been proposed in COVID-19 patients. Ultimately, more well-designed studies with greater sample size, clearly determining issues such as the ideal dosage and intervention timeframe that should be conducted to shed some light on the protective role of vitamin D, especially for those individuals with normal values or population at no risk for COVID-19 [56,85,86,87].

Currently, the potential role of the vitamin B complex in reducing inflammation and breathing difficulties in COVID-19 infected patients has not been proved. For example, no vitamin B deficiency was observed within seven days of admission in 50 patients [52]. Regarding liposoluble vitamins, there was no direct association between vitamin A and E deficiency and COVID-19. Hence, the existing body of knowledge is limited to proposing the adequate intake of these nutrients, but it important to highlight the importance of vitamin A and E in immune system function [88,89]. On the other hand, vitamin C may be beneficial to prevent progression from mild to severe symptoms in COVID-19 patients [90]. A three-day vitamin C supplementation (1 g every 8 h) decreased inflammatory markers in 17 hospitalized patients [91]. A higher dose (6 g every 12 h on day 1 and 6 g for the following four days) reduced the risk of mortality and improved oxygen support in 46 patients [92]. Using bioinformatical network pharmacology, it was proposed the combination of vitamin C and glycyrrhizic acid was a treatment option for COVID-19 [93]. In another case, the co-administration of vitamin C and quercetin, a plant flavonoid found in vegetables, was also proposed [94]. Meanwhile, other studies [95,96] did not provide convincing evidence to support the use of a high dose of vitamin C or in combination with zinc [97] as a treatment option. While currently, large-scale studies are in progress, for example in Canada (LOVIT-COVID) and Italy (NCT04323514), vitamin C intervention strategy is presented as a low-cost promising alternative to improve outcomes in COVID-19 patients. Nevertheless, other related issues such as the severity of illness, the length of the administration, and the ideal dosage should be examined [97].

Vitamin intervention has gained a lot of attention within the scientific community as an effective and low-cost strategy for assisting the immune system to COVID-19. Among the vitamins A to E briefly discussed here, C and D seem to have more evidence supporting their supplementation at an individual level. While ongoing larger-scale COVID-19 studies are expected to clarify the preventive or treatment role of vitamin interventions, especially in high-risk populations. Securing an adequate nutritional status while also considering the recommended upper safety limits, and encouraging an active lifestyle should be a priority of public health policies until a sufficient quantity of vaccines will immunize a large proportion of citizens.

## 6. Dietary Guidelines in COVID-19 Treatments

The European Society for Clinical Nutrition and Metabolism identifies malnutrition to be included in the management of COVID-19 patients as it has a direct impact on health outcomes and incremented healthcare costs [98]. The immune system and antioxidant response are worsened by malnutrition leading to a higher list of complications [99]. Patients with malnutrition are more likely to be from lower socioeconomic groups, and addressing malnutrition is an important step in leaving nobody behind in this fight against the COVID-19 pandemic [100,101]. Some of the symptoms experienced by the COVID-19 patients, such as lack of appetite, loss of taste & smell, fever, or breathing difficulties, among others, affect the nutritional-metabolic pattern. Moreover, isolation, lockdown, and social distancing measures might also drive to limited support for meals.

The systemic inflammation provoked by COVID-19 increases nutritional needs and accelerates muscle loss. Patients admitted to intensive care units also have an increased risk of malnutrition [98], so nutritional medical therapy should be considered as an integral part of the therapeutic approach [26,100,101]. This started at the earliest and no more than 72 h [102]. Associating nutrition to life-support measures has the potential to improve outcomes, particularly in the recovery phase.

The dietary assessment and evaluation of nutritional risks, together with proper risk management, are prudent approaches to deal with the COVID-19 pandemic [26,103]. A well-balanced and diverse diet should ensure optimal intake of all nutrients, especially those that play critical roles in the immune system to help reduce the risk of infections. Non-critically ill patients should follow a healthy diet and supplements, such as vitamins D and E. Long-term patients should not consume hyper nor hypocaloric diets (with caloric ingestion above or below basal metabolism requirements) [104]. In addition, the European Society for Clinical Nutrition and Metabolism guidelines suggest using low carbohydrate diets to avoid insulin resistance and hyperglycemia. High-carbohydrate content (>60%) has also been associated with worsening acute respiratory distress syndrome due to the increase in carbon dioxide production and consequent hypercapnia [102], being the requirement for critically COVID-19 patients 2 g/kg/day and must not exceed 150 g per day [105]. It is also indicated to increase protein supply as top priority complications [99] as patients could experience a loss of up to 1 kg of muscle per day [98,105], to reduce the muscle loss due to the systemic inflammation and enhance the strength of respiratory muscles. The recommendation of proteins is 1.3 g/kg/day increasing the branched-chain amino acids to 50% [105]. The lipid requirement is 1.5 g/kg/day, increasing the proportion of ω-3 fatty acids and ω-9 fatty acids, as well as prioritizing medium and long-chain fatty acids [105,106]. Regarding fluid ingestion is recommended to maintain a neutral fluid balance in critically COVID-19 patients, especially in renal and prerenal failure patients. For stable patients in ICU is recommended 30 mL/kg/day of fluid for adult and 28 mL/kg/day for elderly [105,107].

In line with previous reports about vitamin deficiencies and vitamin intervention, the actual knowledge about supplementation of micronutrients it was highlighted how suboptimal consumption of vitamin B12, vitamin C, vitamin D, and iron is correlated with either COVID-19 incidence or mortality indicators [101,106,107]. Among routine supplementation with multivitamins and minerals, vitamin D deficiency must be assessed [100,101,108,109], as it has been studied that it reduces the risk of the common cold and other similar viral infections [108]. While vitamin C was found to be deficient in COVID-19 patients [110] and could be used to decrease the vulnerability to lower respiratory tract infections, the evidence is still insufficient to support its efficacy to protect people from the SARSCoV2 infection [108,110]. Immunonutrients influence the immune system and improve metabolic and nutritional indices, and can promote patient recovery [104,107,111] by reducing the risk and consequences of infection, including viral respiratory infections [106,107].

Excessive production of pro-inflammatory cytokines increases the risk of a wide range of diseases, including COVID-19, so probiotics and polyphenol supplementation should be carefully assessed [67,105,111]. The increasing prevalence of individuals with malnutrition is related to a greater risk of severe consequences, from COVID-19, in both critically and non-critically ill patients. Further studies should be considered to understand the causal relationships between malnutrition, COVID-19, and metabolic and inflammatory factors. Implementing an optimal nutritional therapy with the right number of macronutrients and with micronutrients and fatty acids supplementation is an essential part of the treatment. Not only for the favorable recovery of COVID-19 patients but also to prevent the deleterious consequences of malnutrition worldwide.

## 7. Nutrition, Immunology and COVID-19

To date, despite the existence of several vaccines in motion, the lack of logistics and production means that the global population must learn to live for a longer time with the virus among us. Therefore, we must continue to rely on our natural defense system to deal with the SARS-CoV-2. Given that the virus, as well as its affection, is multi-organ, and therefore, different organ systems are affected in the immune response process, the greater the protective factors, the better the response and the prognosis of the infected person. Thus, we discuss the effect of nutrition on different systems and its interaction with the SARS-CoV-2 infection.

### 7.1. Immunonutrition as a Cornerstone

Clinical nutrition plays an important role, and above all, is essential for the multidisciplinary patient management affected by the known SARS-CoV-2 [112]. It is even important for those patients who have not contracted the disease but have a pathological history of cardiovascular disease, diabetes mellitus, or poor metabolic control since these may worsen the affection of the virus [113]. Yet, independently of the previous clinical history of the patient, all subjects have the same risk of contracting the SARS-CoV-2 [114]. However, subjects with pathological history have an increased risk of mortality, mainly due to the response to inflammatory disease generated by the immune system [114]. This severe immune response is given by multiple factors, one of them is the degree of previous inflammation that the organism has, and as a result of this, premature senescence of the immune system [114]. However, the state of chronic inflammation of the organism can be prevented by changes in the lifestyle, such as appropriate nutrition [115], the correct amount of physical exercise [7,116], and good mental state/health [3]. Hence, nutrition has undoubtedly an essential role in responding to the disease and specifically “immunonutrition”, a cornerstone in understanding the inflammatory response, either as a prevention or treatment factor. Immunonutrition is an emerging and interdisciplinary subject since it covers different aspects related to Nutrition, Immunity, Infection, Inflammation, and Injury or tissue damage. Different interactions are made between the endocrine, nervous and immune systems, being the latter part, the gut microbiome. Furthermore, these systems are highlighted and affected by the novel SARS-CoV-2.

### 7.2. Endocrine System, SARS-CoV-2 & Nutrition

The endocrine glands’ diseases highlight the importance of hormonal and nutritional factors in the regulation of metabolism. Nutritional alterations affect each aspect of the functioning of the endocrine glands leading to serious disorders. Thus, nutrition and endocrinology are linked with the premise that adequate nutrition is essential for the maintenance of organism balance and homeostasis [117].

Furthermore, hormones could affect the phenotype, typically of behavior, as well as regulating development, growth, reproduction, metabolism, and immunity [118]. The abundance of hormone receptors themselves can explain differences between phenotypes among individuals when encountering specific stressors [119], which may be a plausible explanation for the “cytokine storm” (severe immune reaction in which the body releases too many cytokines into the blood too quickly) seen among different subjects infected with SARS-CoV-2. Furthermore, the pathogenesis of COVID-19 entails entry of SARS-CoV-2 via the respiratory system and lodgment in the lung parenchyma. Notably, the angiotensin-converting enzyme-2 receptor is the entry route of coronaviruses to the host cell, widely expressed in the endocrine organs, including testis, endocrine pancreas, thyroid, and adrenal, and pituitary glands [120]. Highlighting the importance of this system on the SARS-CoV-2 response.

### 7.3. Immune System SARS-CoV-2 & Nutrition

The immune system responds to the SARS-CoV-2 via a cytokine storm and hyper inflammation, which itself leads to further multi-organ damage and, in the worst scenario, to death [121]. Yet, it is a fact that people consuming a well-balanced diet are healthier, with a strong immune system, and present a reduced risk of chronic illness, infectious diseases [121]. Furthermore, the immune system is always active, which is accompanied by an increased metabolism rate, requiring energy sources, substrates for biosynthesis, and regulatory molecules. These energy sources, substrates, and regulatory molecules are ultimately derived from the diet. Hence an adequate supply of a wide range of nutrients is essential to support the immune system to function optimally [122].

In this line, the bibliography suggests that vitamins from group B are essential in viral and bacterial infections [123]. Vitamin C is considered as a protective from flu-like symptomatology [124]. Vitamins E and D among zinc have been found essential for the immune system and are especially being studied in the COVID-19 [112]. High protein consumption (>15%) may be a top priority since it induces immunoglobulin production and potential antiviral activity [125]. Furthermore, recent studies suggest that in a regular meal, individuals should eat fruit, vegetables, legumes, nuts, whole grains, unsaturated fats, white meats, and fish. Fruit juice, tea, and coffee can also be consumed cautiously since, sweetened fruit juices, fruit juice concentrates, syrups, fizzy drinks, and still drinks must be avoided [126]. Saturated fat, red meat, more than 5 g salt per day, and industry processed food should be avoided [127].

### 7.4. Nervous System, SARS-CoV-2 & Nutrition

There were reported neurological manifestations and complications of the SARS-CoV-2, suggesting that it could reach the peripheral nervous system and central nervous system since patients suffer from neurological manifestations as anosmia, confusion, and ageusia, and viral particles have been specifically documented in endothelial cells of the kidney, lung, skin, and central nervous system. This, meaning that the blood endothelial barrier may be considered as the main route for SARS-CoV-2 entry into the nervous system, with the barrier disruption being more logical than barrier permeability, as evidenced by postmortem analyses [128].

The blood endothelial barrier is a dynamic and complex interface between the blood and the central nervous system regulating brain homeostasis. Bibliography suggests that the penetration of neuroprotective nutrients, especially plant polyphenols and alkaloids, may have a potential protective effect on brain endothelium [129]. Furthermore, the neuroprotective effects of extracts and constituents of medical plants, spices, and dietary supplements were demonstrated in both preclinical experiments and clinical trials [130].

In this line, the following nutrients and nutraceuticals have been found to offer a somewhat brain-protective effect [131]. Lipids as Omega-3 fatty acid (Eicosapentaenoic, Docosahexaenoic, Linoleic α-lipoic acids), vitamins (C, B9, D3, and E), Plant polyphenols (Flavone as Apigenin and Luteolin; Flavonol as Tangeretin, Chrysin, Quercetin; Isoflavone as Naringenin, Naringin, Hesperetin, Rutin; Antocyanidines; Phenolic Acids; Stilbene; Trace elements as Theophylline, Capsaicin, Piperine, and Zin; Endogenous antioxidants as Selenium, Glutathione, Melatonin, Creatin, and N-acetyl-cysteine). However, these dietary compounds need to reach effective concentrations in the central nervous system to exert. Yet, knowledge is still scarce and continuously growing due to novel technologies being an area of intense research.

### 7.5. Gut Microbiome, SARS-CoV-2 & Nutrition

There are approximately 100 trillion microorganisms within the gut, tenfold greater than the number of cells in the human body. It is in contact with the body’s immune cells and with the second largest pool of neural cells in the body, the largest being located in the brain [132]. This large pool of microorganisms residing on mucosal surfaces of the gastrointestinal tract has both direct and indirect effects upon the host immune system, being an estimated 70% of the immune system response located within the gastrointestinal tract [133]. Given the importance of the intestinal microbiota in the immune response and knowing that SARS-CoV-2 progression appears to be associated with the “cytokine storm” which leads to hyper-inflammation (elevated levels of pro-inflammatory cytokines, including TNFα, IL-6, and IL-1β), a special focus should be given.

Actual authors have tried to address the mechanism whereby the gut microbiota may facilitate or difficult the viral transmission of SARS-CoV-2. Since COVID-19 RNA has been found in feces [134], there is a door open for a new link. Recent authors found increased levels of *Clostridium ramosum* and *Clostridium hathewayi*, which are associated with severity of SARS-CoV-2 symptomatology along with reduced levels of *Alistipes* spp [135]. Furthermore, *Coprobacillus* spp. has been linked to the regulation of ACE2 in the murine gut [136], being the ACE2 a transmembrane protein that counteracts ACE, which receptors are found within epithelium cells of the gut. Thus, changes in the gut microbiota may alter the ability of the virus to gain cellular entry into the gut [137]. Other studies support Zuo’s findings, detecting more potential pathogens in the gut microbiota of 30 SARS-CoV-2 hospitalized patients [138].

Furthermore, recent studies found how the gut composition is significantly altered in COVID-19 patients independently of whether patients had received medication [139]. Authors suggested that reinforcing beneficial gut species depleted in COVID-19 could serve as a novel way to mitigate severe disease, underscoring the importance of managing patients’ gut microbiota during and after COVID-19 [139]. These changes in the microbial profile after the pathogenesis of the COVID-19 were also found in patients during the hospitalization, suggesting an early affection of the microbial profile [140]. Despite this, more studies are necessary, as it seems that the gut microbiome is involved in the magnitude of COVID-19 severity possibly via modulating host immune responses. Thus, for anticipatory purposes, diagnosing gastrointestinal symptoms that precede respiratory problems during COVID-19 could be necessary to improve early detection and treatment. Further research should assess the composition of the gut microbiota and its metabolic products in the context of COVID-19, which could help to determine new biomarkers of the disease, helping identify new therapeutic targets [141].

Despite the intestinal microbiota and its composition depends on genetic and epigenetic factors [142], nutrition has shown to be a factor that is capable of acutely modifying its composition [143]. Interventions have been shown to have a positive and significant effect on human health [144]. In addition, dietary supplements like fiber and probiotics have been shown to improve microbial derangements in health [145], but the precise mechanisms of how nutrition and dietary supplements modulate the gut microbiome remain to be determined.

## 8. Physical Activity and COVID-19

Previous authors have shown how older patients, with comorbidities, cardiovascular risk factors, and systemic diseases have a poorer prognosis with the coronavirus infection [146,147]. In the absence of actual effective treatment, the control of these pathologies is basic and essential. In this line, physical activity has been shown a positive effect on these diseases and a decrease in all-cause mortality [148,149]. Specifically, COVID-19 patients are characterized by a large inflammatory response [150], hypoxemia [151], impaired respiratory function [152], where the angiotensin-converting enzyme 2 has been proposed as the receptor for SARS-CoV-2 protein in the alveolar epithelial cells in the lungs [153], all these factors associated with critical and fatal illnesses. In this line, exercise was associated with decreases in inflammatory markers [154], decreases in basal minute ventilation and increased oxygen uptake [155], a reduced upper respiratory tract infections incidence and duration [156], and a shift in the renin-angiotensin system towards angiotensin 1–7 which may reduce the severity of the clinical outcome of COVID-19 infection [157].

Several authors highlighted the importance of metabolic health as a modulator of illness severity, emphasizing how metabolic complications related to inactivity and obesity were fully operating to favor COVID-19 diffusion [158]. In this line, it was shown how weaker and debilitated muscle strength was associated with a higher risk of severe COVID-19 [159]. The association between muscle strength and COVID-19 severity is related to the essential role of muscle in health and disease [160]. Poor skeletal muscle functions negatively affect the immune response, motor function, respiratory function, and metabolic stress when facing acute infection [161,162]. All these factors have been previously identified as a COVID-19 severity modulator [163].

Cardiorespiratory fitness has been proposed to be beneficial in COVID-19 since it allows a greater inflammatory response control and potentially enhancing antiviral host responses following infection [164]. In this line, higher cardiorespiratory fitness levels are related to a lower hospitalization due to COVID-19 [165]. It is known how physical activity modulates immune system functions, presenting moderate physical activity an association with cardiorespiratory fitness, increasing immune system capacity, and reducing inflammation [166]. In this regard, a sedentary lifestyle was found as an independent risk factor for mortality in COVID-19 hospitalized patients. This fact represents an important finding and proposes the utility of exercise in the prevention of severe COVID-19 presentations [167]. In addition, the walking pace has been identified as a potential risk factor for severe COVID-19, with slow walkers having a high-risk profile [168]. Physical activity and cardiorespiratory fitness have clear preventive potential on several chronic diseases that are considered to be risk factors for COVID-19 outcomes and counteract aging-related processes that may also be associated with higher COVID-19 risk [169]. Maximal exercise capacity is independently and inversely associated with the likelihood of hospitalization due to COVID-19.

In the actual situation, where vaccines are starting to administrate to the population, also physical exercise could have a critical role. Previous authors demonstrated how an acute stressor in close temporal proximity to immune challenge could enhance the response to delayed-type hypersensitivity and antibody response to vaccination [170]. Specifically, acute exercise-induced stress before influenza vaccination (preferably high-intensity exercise) might enhance antibody responses [171,172]. Beyond vaccination, the COVID-19 pandemic has taught us the importance of preventive lifestyle actions. Physical activity is presented as a safe and potential preventive measure, especially for the most vulnerable groups [173].

Finally, given the ongoing novelty of COVID-19, some authors proposed the evaluation of both inflammatory response and physical function (handgrip strength) for patients recovering from COVID-19 since it provides new information into the recovery process [174]

## 9. Practical Statements

The main findings of the present and their practical applications are summarized in the following key points and Table 1.

- The COVID-19 lockdown promoted unhealthy dietary changes (inactivity, daily intake, snacks, alcohol), increasing body mass and fat, and showing obesity-overweight people poor diet habits.
- Obesity is a risk factor for COVID-19.
- A healthy balanced diet is an integral part of personal risk management.
- Vitamins C and D improve health-related outcomes in COVID-patients.
- Sufficient vitamin intake and an active lifestyle are strongly recommended as a preventive measure to the general population.
- There is a large prevalence of malnutrition among hospitalized patients with COVID-19.
- Nutritional support and rehabilitation exercise are needed to avoid muscle atrophy and sarcopenia in COVID-19 hospitalized patients. They should be considered as an integral part of the therapeutic approach.
- Deficient states of vitamin C, D, B12 selenium, iron, ω-3, and medium and long-chain fatty acids increase the probability of hospitalization and mortality from COVID-19.
- The gut microbiome profile is altered due to COVID-19, being involved in the magnitude of COVID-19 severity via modulating host immune responses.
- A healthy gut microbiome serves as a preventive and protective factor, appropriate nutrition and probiotics are good strategies for its enhancement.
- Active lifestyle and physical activity allow a lower risk, and mortality rate in COVID-19 patients, due to its positive effect on metabolic health and inflammation.

Finally, the present review has certain limitations. First, the information regarding the study aim has been presented with a low number of studies compared to traditional reviews. This fact was due to the novelty of the review and the relatively short time since the appearance of COVID-19. Based on the results presented, we would propose as future research lines in this area the study of the effect of diets with a powerful anti-inflammatory effect in patients with COVID-19 and the risk of COVID-19 infection; and to analyze the effect of combinations of supplements with antioxidant and anti-inflammatory effects in patients with COVID-19.

## 10. Conclusions

The present narrative review found how the COVID-19 lockdown promoted unhealthy dietary changes and increases in body weight of the population, showing the obesity and physical activity levels as risk factors for COVID-19. In addition, hospitalized COVID-19 patients presented malnutrition and deficiencies in vitamin C, D, B12 selenium, iron, ω-3, and medium and long-chain fatty acids highlighting the potential health effect of vitamin C and D interventions.

## Figures and Tables

**Table 1:** Nutritional interventions in COVID-19.

Recommendation	Nutritional Intervention	
Avoid	Daily products	
	Snacks	
	Alcohol	
Include	Carbohydrates	<60 % of total caloric value to avoid insulin resistance, hyperglycemia, and acute respiratory distress syndrome. 2 g/kg/day and must not exceed 150 g/day for critically COVID-19 patients.
	Proteins	1.3 g/kg day to reduce muscle loss due to systemic inflammation and improve respiratory muscle strength.
	Fats	1.5 g/kg/day
	Fluids	For stable patients in ICU: 30 mL/kg/day of fluid for adult and 28 mL/kg/day for elderly
Prevent Deficient states	Vitamin C	
	Vitamin D	
	Vitamin B12	
	Selenium	
	Iron	
	ω-3, and medium and long-chain fatty acids	
Keep	Adequate gut microbiome profile	
**Physical Activity Intervention**		
Avoid	Inactivity	
Keep	Active lifestyle	

## Data Availability

Not applicable.
